# Association between sleep-wake schedules and myopia among Chinese school-aged children and adolescents: a cross-sectional study

**DOI:** 10.1186/s12886-023-02874-9

**Published:** 2023-04-03

**Authors:** Shaojun Xu, Zhiqiang Zong, Yi Zhu, Xindong Zhang, Yi Zhang, Xi Wang, Shuman Tao, Xiaoyan Wu, Fangbiao Tao

**Affiliations:** 1 Department of Maternal, Child & Adolescent Health, School of Public Health, Anhui Medical University, 230032 Hefei, Anhui China; 2 MOE Key Laboratory of Population Health Across Life Cycle (Anhui Medical University), 230032 Hefei, Anhui China; 3 Anhui Provincial Key Laboratory of Population Health & Aristogenics, Anhui Medical University, 230032 Hefei, Anhui China; 4 The Second School of Clinical Medicine, Anhui Medical University, 230032 Hefei, Anhui China; 5 Baoan District Center for Disease Control and Prevention, 518101 Shenzhen, Guangdong China; 6 Department of Ophthalmology, The Second Hospital of Anhui Medical University, 230601 Hefei, Anhui China

**Keywords:** Sleep hygiene, Circadian rhythm, Myopia, Child, Adolescent

## Abstract

**Background:**

This study aimed to investigate the relationship between sleep-wake schedules and self-reported myopia in the pediatric population.

**Methods:**

In this cross-sectional study in 2019, school-aged children and adolescents in the Baoan District of Shenzhen City were sampled using a stratified cluster sampling approach. Sleep-wake schedules of children were determined by a self-administered questionnaire. The age that participants first reported using myopia correction glasses or contact lenses was used to identify those with myopia. Pearson χ^2^ test was used to examine differences in myopia prevalence among participants with different characteristics. Multivariate logistic regression, adjusted for potential confounding variables, was applied to examine the relationship between sleep-wake schedule and risk of self-reported myopia, and a stratification analysis by school grade was also performed.

**Results:**

A total of 30,188 students were recruited. In this study, the overall prevalence of myopia was 49.8%, with prevalence rates of 25.6%, 62.4%, and 75.7% for primary, junior high, and senior high school students, respectively. Students with irregular sleep-wake times reported a higher prevalence of myopia than those with regular sleep-wake times. Nighttime sleep duration of < 7 hours/day (h/d) (OR = 1.27, 95%CI: 1.17–1.38), no daytime nap (OR = 1.10, 95%CI: 1.03–1.18), irregular weekday bedtime (OR = 1.11, 95%CI: 1.05–1.17), irregular weekday wake time (OR = 1.21, 95%CI: 1.12–1.30), weekend bedtime delayed > = 1 h/d (OR = 1.20, 95%CI: 1.11–1.29, P < 0.001), weekend wake time delayed > = 1 h/d (OR = 1.11, 95%CI: 1.03–1.19), irregular sleep-wake time on weekdays (OR = 1.13, 95%CI: 1.07–1.19), and social jetlag > = 1 h (OR = 1.08, 95%CI: 1.03–1.14) were likely to be associated with increased risks of self-reported myopia after adjusting age, sex, grade, parental education level, family income, parental myopia, academic record, and academic workload. When stratified by school grade, we observed that nighttime sleep duration < 7 h/d, no daytime naps, and irregular sleep-wake time on weekdays were significantly associated with self-reported myopia in primary school students.

**Conclusion:**

Insufficient sleep and irregular sleep-wake schedules can increase the risk of self-reported myopia in children and adolescents.

## Background

Myopia has developed into a major public health issue that has a negative impact on the health of children and adolescents worldwide [1]. By 2050, it is predicted that the prevalence of myopia and high myopia will increase significantly, affecting up to 5 billion and 1 billion individuals, respectively [2]. Severe myopia-related ocular diseases, including cataracts, glaucoma, retinal detachment, and myopic maculopathy, can result in irreversible vision loss, which significantly negatively impacts one’s quality of life [3]. Developing myopia increases children’s and adolescents’ risk of developing high and extreme myopia [4]. Therefore, investigating potential risk factors to develop more effective prevention strategies for myopia in the pediatric population is urgent and critical.

The sleep-wake schedule is regulated by the circadian system [5]. Irregular sleep-wake schedules reflect circadian rhythm sleep-wake disorders [6]. Previous studies have reported that irregular sleep-wake schedules and chronic lack of sleep duration were generally associated with adverse outcomes in academic or occupational performance, economic status, mental health, and social connections [7–9]. Recently, insufficient sleep and irregular sleep-wake schedules are common among schoolchildren and adolescents. A cross-sectional study in South Korea with 3,625 participants aged 12 to 19 observed that refractive error increased by 0.10 D for every one-hour decrease in sleep duration [10]. Another study found that children’s sleep-wake patterns varied significantly on weekdays and weekends, and myopic children tended to have shorter sleep latency than non-myopic ones [11]. Similarly, Liu et al. found that late bedtime and late wake-up time were associated with baseline myopia in school-aged children [12]. These observational studies suggest a complex relationship between sleep duration, sleep-wake schedule, and myopia risk in the pediatric population. Therefore, this cross-sectional study was conducted to test the hypothesis that insufficient sleep duration and irregular sleep-wake schedules are associated with myopia risk in children and adolescents.

### Methods

#### Participants

Participants were sampled from 1st to 12th-grade students from 14 schools in 6 sub-districts (Xixiang, Songgang, Shiyan, Fuyong, Shajing, and Xinan) of Baoan District of Shenzhen City using a stratified cluster sampling approach from April to May 2019. The total number of students was 33 801, and 30 188 valid questionnaires were received, with a response rate of 89.3%. Finally, 13 420 (44.45%) students in grades 1 to 6 (mean age = 9.16 years, SD = 1.87), 8 232 (27.27%) students in grades 7 to 9 (mean age 13.54 years, SD = 1.02), and 8 536 students (28.28%) in grades 10 to 12 (mean age 16.54 years, SD = 1.10) were analyzed. The study was approved by the Institutional Ethics Committee of Anhui Medical University (2140104) and adhered to the tenets of the Declaration of Helsinki. Written informed consent of parents was obtained from all participants under 18 years of age for the study.

### Data collection

Students in grades 5 to 12 or their parents/guardians (for students in grades 1 to 4) were asked to complete a self-administered questionnaire. We collected basic information as follows: sex (male/female), grade (primary/junior high/senior high), family income (low/middle/high), father’s education level (junior high or below/senior high/college or above), mother’s education level (junior high or below/senior high/college or above), academic record (bad/normal/good), academic workload (light/normal/heavy); genetic factors: presence of myopia in father (yes/no/unknown) and mother (yes/no/unknown).

Participants were asked about the actual night sleep duration and daytime nap duration after lunch in the last month. Nighttime sleep duration was divided into three categories (< 7 h/d, 7–8 h/d, >= 8 h/d) by the National Sleep Foundation’s sleep duration recommendations [13]. Considering a 37 ~ 53 min nap to be healthy for teenagers, daytime napping duration was classified into three groups: none, < 0.5 h/d, and > = 0.5 h/d [14]. In the following analysis, regular weekday bedtime refers to children going to bed at a consistent or fixed time during the weekday. Similarly, regular weekday wake time refers to children waking up at a consistent or fixed time during the weekday. The inability to stay at a consistent or fixed time to sleep/wake up is considered irregular. According to the National Sleep Foundation poll in 2019 [15], weekend bedtime and weekend wake time were classified into three items: did not deviate, delayed < 1 h/d, and delayed > = 1 h/d. Regular sleep-wake time on weekdays means that both bedtime and wake time are consistent as usual, while irregular refers to changes in bedtime or wake time, with at least one being inconsistent. Social jetlag refers to the discrepancy between sleep time on work and free days, or weekdays and weekends, and is assumed to reflect the difference between social and biological time [16]. In previous studies on social jetlag, a one-hour difference was used as the defining criterion, and less than one hour was considered no social jetlag [17, 18]. In this study, social jetlag was classified into two items: <1 h and > = 1 h.

Children and adolescents were classified as self-reported myopia if they wore myopia-correcting glasses or contacts or provided information about the age at which they first started wearing them. Wearing corrective glasses for hyperopia (farsightedness) is not classified as self-reported myopia. This method of myopia determination has also been used in other high-quality studies [19, 20]. Moreover, Cumberland et al. have demonstrated that self-reported history of using optical correction can accurately identify myopia [21].

### Statistical analysis

The Statistical Package for Social Science (SPSS) version 23.0 (IBM Corp., Armonk, NY) was used to analyze all the data. We first conducted the Pearson χ^2^ test to examine differences in myopia prevalence among children and adolescents with different characteristics. Then, multivariate logistic regression was performed to examine the associations of myopia with nighttime sleep duration, daytime napping, and sleep-wake schedule. Finally, we performed stratification analysis according to school grade (primary school, junior high school, and senior high school) and sleep-wake time on weekdays (regular/irregular). In this study, the odds ratio (OR) and 95% confidence interval (95% CI) were used to describe the strength of the association. CI that did not contain an OR of 1 was considered significant. The level of statistical significance for a test of the hypothesis was set at 5%.

## Results

### Baseline Characteristics and prevalence of self-reported myopia

Table 1 presents the baseline characteristics of participants. 49.8% of the participants reported myopia. The prevalence of self-reported myopia is higher in girls than in boys. According to the χ^2^ trend test, the myopic prevalence increased with grade level. We also found that higher family income, lower parental education level, and parental myopia were risk factors for self-reported myopia. Moreover, the results showed that the prevalence of myopia was higher among students with good academic records and heavy academic workloads. The prevalence of myopia in school-aged children increased with less nighttime sleep duration. Students with irregular weekday bedtimes had a higher prevalence of self-reported myopia than those with regular weekday bedtimes. Children and adolescents with irregular weekday wake times had a higher prevalence of myopia than those with regular weekday wake times. The prevalence of self-reported myopia increased as weekend bedtimes and weekend wake times became more delayed.

## Association between sleep duration, sleep-wake schedule, and self-reported myopia

The associations between sleep duration, sleep-wake schedule, and self-reported myopia are presented in Table 2. Age, sex, grade, parental education, family income, parental myopia, academic record, and academic workload were all significantly associated with the prevalence of self-reported myopias, and they were adjusted for the main model. The model was also used in all subsequent models. After adjusting for all confounders, having nighttime sleep duration of < 7 h/d and no daytime naps were significantly associated with self-reported myopia. School-aged children and adolescents with irregular weekday bedtimes and wake times, weekend bedtimes and wake times delayed > = 1 h/d, irregular sleep-wake times on weekdays, and social jetlag had a higher risk of self-reported myopia.

The associations stratified by school grade were performed (Table 3). After adjusting for potential confounders, nighttime sleep duration < 7 h/d was associated with self-reported myopia. No daytime naps, irregular weekday bedtimes, irregular weekday wake times, delayed weekend bedtimes, delayed weekend wake times, irregular sleep-wake times on weekdays, and social jetlag > = 1 h were associated with an increased risk of self-reported myopia among primary school students. Among children and adolescents who had regular sleep-wake times on weekdays, those with shorter nap duration (none and < 0.5 h/d), shorter nighttime sleep duration (< 7 h/d and 7–8 h/d), delayed weekend bedtimes ( > = 1 h/d) and delayed weekend wake times ( > = 1 h/d) were found to have an increased risk of self-reported myopia (Table 4).

## Discussion

Insufficient sleep and myopia in children and youth are recognized as major public health issues [22]. The crucial role of sleep in physical and mental health is well known, especially during the developmental period. In recent years, there has been growing interest in examining the relationship between sleep patterns and myopia in children and adolescents. To the best of our knowledge, this is the first cross-sectional study to examine the association between sleep duration, sleep-wake schedule, and risk of self-reported myopia in school-aged children and adolescents.

A healthy sleep schedule needs to satisfy the following conditions: it should have sufficient duration, be at a proper and regular timing, of good quality, and the person needs to have no sleep disorder or other factors that present an obstacle to sleep. Members of the American Academy of Sleep Medicine issued consensus recommendations that adolescents aged 6–12 years old should sleep 9–12 h/d and 13–18 years old should sleep 8–10 h/d regularly to promote the best health [23]. Our findings suggest that shorter sleep duration (< 7 h/d) and irregular sleep-wake schedules are significantly associated with the risk of self-reported myopia in pediatric and adolescent populations. Humans live in a diurnal world with a 24-hour cycle of light and darkness. Organisms can synchronize their circadian rhythms and sleep-wake cycles to the appropriate temporal niche through the daily light-dark cycle. In simplified terms, bright morning light advances the circadian rhythm, while night light delays it [24]. The phase of peripheral tissue clocks and pacemaker neural clocks is altered by light exposure at incorrect times of the day [25]. Over the past 100 years, natural light patterns have been significantly disrupted by the introduction of artificial light into the night environment [26]. In our modern society, many students are exposed to indoor light instead of having outdoor activities in natural sunlight. During the day, the indoor environment is relatively dark. However, at night, artificial lighting not only increases the intensity of light above natural nighttime but also reduces the duration of darkness, which puts humans out of sync with natural circadian rhythms. An observational study showed that using objective indicators in children could observe significant diurnal changes in multiple eye and systemic parameters within 24 hours, and the rhythms of the eye axis and choroidal thickness were approximately opposite [27]. An animal experiment revealed that exposure to midnight light altered the rhythm of the ocular axis and choroidal thickness, which may be associated with the occurrence of refractive error in the eyes [28].

Previous epidemiological studies have also examined the association between disordered sleep and myopia in children.

According to population-based epidemiological evidence by Jee et al., myopia and sleep duration have an inverse association, while high myopia is unrelated to sleep duration [10]. Ayaki et al. reported that sleep quality in Japanese children was significantly associated with myopia, especially in the high myopia group [29]. In Ayaki’s study, substantial correlations were observed between high myopia and low Pittsburgh Sleep Quality Index (PSQI) scores, little sleep time, and late bedtime. In contrast, a cross-sectional study conducted by Zhou et al. reported that the association between total sleep duration (night + midday) and myopia risk was not significant (*P* = 0.199) in Chinese children [30]. School schedules may interfere with sleep schedules and increase the risk of developing myopia, as more school time appears to be a risk factor for myopia [31, 32]. In addition, information on other potential determinants of myopia, such as parental myopia, was not available in Zhou’s study, and this may influence the results of multivariate logistic regression, resulting in a weak association. In another cross-sectional study conducted in Shanghai, Qu et al. found that myopia was not statistically associated with sleep duration during the semester and holidays [33]. This may be related to myopic participants not having high myopia and not having a corrected visual acuity < 1.0. Also, since the data of Qu’s study are based on the findings of a few schools in a suburban area, the results may not be universally representative. In

**Table 1:** Characteristics of the full sample and prevalence of self-reported myopia.

Variables	Number of participants	Self-reported myopia cases	Self-reported myopia rates (%)	*χ*^2^ or *χ*^2^-trend	*P* value
Age				5302.51^a^	< 0.001
<= 12 years	14,484	4,049	28.0		
> 12 years	15,704	10,977	69.9		
Sex				190.00^a^	< 0.001
Male	16,897	7,816	46.3		
Female	13,291	7,210	54.2		
Grade				5958.23^b^	< 0.001
Primary	13,420	3,431	25.6		
Junior high	8,232	5,137	62.4		
Senior high	8,536	6,458	75.7		
Family income				1127.34^b^	< 0.001
Low	4,113	1,598	38.9		
Middle	20,395	10,494	51.5		
High	5,680	2,934	51.7		
Father’s education				52.49^b^	< 0.001
Junior high or below	9,641	5,025	52.1		
Senior high	11,387	5,712	50.2		
College or above	9,160	4,289	46.8		
Mother’s education				119.86^b^	< 0.001
Junior high or below	12,203	6,448	52.8		
Senior high	11,184	5,554	49.7		
College or above	68,01	3,024	44.5		
Father myopia				442.40^a^	< 0.001
Yes	8,414	4,936	58.7		
No	20,791	9,503	45.7		
Unknown	983	587	59.7		
Mother myopia				290.62^a^	< 0.001
Yes	8,696	4,955	57.0		
No	2,0524	9,525	46.4		
Unknown	968	546	56.4		
Academic record				148.18^b^	< 0.001
Bad	7,580	3,494	46.1		
Normal	11,573	8,627	49.1		
Good	5,035	2,905	57.7		
Academic workload				397.40^b^	< 0.001
Light	2,451	1,035	42.2		
Normal	19,020	8,847	46.5		
Heavy	8,717	5,144	59.0		
Nighttime sleep duration					
< 7 h/d	13,354	9,162	68.6	3754.13^b^	< 0.001
7–8 h/d	7,862	3,395	43.2		
>= 8 h/d	8,972	2,469	27.5		
Daytime nap duration				616.41^a^	< 0.001
None	6,022	2,482	41.2		
< 0.5 h/d	9,229	5,542	60.0		
>= 0.5 h/d	14,937	7,002	46.9		
Weekday bedtime				198.59^a^	< 0.001
Regular	19,585	9,164	46.8		
Irregular	10,603	5,862	55.3		
Weekday wake time				122.67^a^	< 0.001
Regular	25,515	12,352	48.4		
Irregular	4,673	2,674	57.2		
Weekend bedtime				119.98^b^	< 0.001
Did not deviate	6,263	2,945	47.0		
Delayed < 1 h/d	14,439	6,852	47.5		
Delayed > = 1 h/d	9,486	5,229	55.1		
Weekend wake time				309.10^b^	< 0.001
Did not deviate	5,610	2,400	42.8		
Delayed < 1 h/d	11,793	5,519	46.8		
Delayed > = 1 h/d	12,,785	7,107	55.6		
Sleep-wake time on weekdays				235.61^a^	< 0.001
Regular	18,526	8,572	46.3		
Irregular	11,662	6,454	55.3		
Social jetlag				125.46^a^	< 0.001
< 1 h	12,088	5,540	39.0		
>= 1 h	18,100	9,486	50.9		

Note: ^a^ is for *χ*^2^, ^b^ is for *χ*^2^ or *χ*^2^-trend.

**Table 2:** Association between sleep-wake schedule and self-reported myopia in children and adolescents.

Variables		OR (95%CI)	*P* value
Nighttime sleep duration			
< 7 h/d		1.27 (1.17–1.38)	< 0.001
7–8 h/d		1.15 (1.07–1.27)	< 0.001
>= 8 h/d		1.00	
Daytime nap duration			
None		1.10 (1.03–1.18)	0.005
< 0.5 h/d		1.06 (0.99–1.12)	0.056
>= 0.5 h/d		1.00	
Weekday bedtime			
Regular		1.00	
Irregular		1.11 (1.05–1.17)	< 0.001
Weekday wake time			
Regular		1.00	
Irregular		1.21 (1.12–1.30)	< 0.001
Weekend bedtime			
Did not deviate		1.00	
Delayed < 1 h/d		1.08 (1.01–1.16)	0.029
Delayed > = 1 h/d		1.17 (1.09–1.25)	< 0.001
Weekend wake time			
Did not deviate		1.00	
Delayed < 1 h/d		1.05 (0.97–1.13)	0.207
Delayed > = 1 h/d		1.11 (1.03–1.19)	0.006
Sleep-wake time on weekdays			
Regular		1.00	
Irregular		1.13 (1.07–1.19)	< 0.001
Social jetlag			
< 1 h		1.00	
>= 1h		1.08 (1.03–1.14)	0.003

Note: adjusted for age, sex, family income, parental education level, parental myopia, academic record, and academic workload.

**Table 3:** Association between sleep-wake schedule and self-reported myopia according to school grade.

Variables	Primary school			Junior high school			Senior high school	
	*OR* (95%*CI*)	*P* value		*OR* (95%*CI*)	*P* value		*OR* (95%*CI*)	*P* value
Nighttime sleep duration								
< 7 h/d	1.20 (1.05–1.38)	0.008		1.18(1.02–1.36)	0.029		1.82 (1.28–2.59)	0.001
7–8 h/d	1.04 (0.95–1.14)	0.431		1.05 (0.91–1.22)	0.493		1.53 (1.05–2.23)	0.028
>= 8 h/d	1.00			1.00			1.00	
Daytime nap duration								
None	1.16 (1.06–1.28)	0.002		0.93 (0.83–1.05)	0.240		0.95 (0.75–1.21)	0.670
< 0.5 h/d	1.06 (0.95–1.19)	0.309		1.04 (0.93–1.15)	0.487		1.02 (0.92–1.13)	0.718
>= 0.5 h/d	1.00			1.00			1.00	
Weekday bedtime								
Regular	1.00			1.00			1.00	
Irregular	1.22 (1.11–1.33)	< 0.001		0.96 (0.87–1.05)	0.340		0.94 (0.84–1.04)	0.230
Weekday wake time								
Regular	1.00			1.00			1.00	
Irregular	1.25 (1.11–1.41)	< 0.001		1.14 (1.01–1.30)	0.034		1.12 (0.98–1.27)	0.094
Weekend bedtime								
Did not deviate	1.00			1.00			1.00	
Delayed < 1 h/d	1.15 (1.03–1.27)	0.011		1.09 (0.96–1.22)	0.182		1.03 (0.90–1.19)	0.664
Delayed > = 1 h/d	1.26 (1.11–1.42)	< 0.001		1.16 (1.02–1.31)	0.024		1.02 (0.89–1.18)	0.752
Weekend wake time								
Did not deviate	1.00			1.00			1.00	
Delayed < 1 h/d	1.09 (0.98–1.21)	0.125		1.13 (0.99–1.28)	0.073		0.93 (0.78–1.10)	0.372
Delayed > = 1 h/d	1.18 (1.05–1.32)	0.004		1.07 (0.95–1.22)	0.243		0.99 (0.85–1.17)	0.974
Sleep-wake time on weekdays								
Regular	1.00			1.00			1.00	
Irregular	1.23 (1.12–1.34)	< 0.001		0.97 (0.89–1.06)	0.528		0.97 (0.88–1.08)	0.617
Social jetlag								
< 1 h	1.00			1.00			1.00	
>= 1 h	1.11 (1.02–1.21)	0.012		1.06 (0.96–1.16)	0.260		1.06 (0.95–1.19)	0.274

Note. : adjusted for age, sex, family income, parental education level, parental myopia, academic record, and academic workload.

**Table 4:** Association between nighttime sleep duration, daytime napping, and self-reported myopia in children and adolescents with regular sleep-wake time on weekdays.

Variables		OR (95%CI)	*P* value
Nighttime sleep duration			
< 7 h/d		1.32 (1.19–1.48)	< 0.001
7–8 h/d		1.18 (1.07–1.28)	< 0.001
>= 8 h/d		1.00	
Daytime nap duration			
None		1.11 (1.01–1.22)	0.028
< 0.5 h/d		1.09 (1.01–1.18)	0.037
>= 0.5 h/d		1.00	
Weekend bedtime			
Did not deviate		1.00	
Delayed < 1 h/d		1.06 (0.97–1.15)	0.216
Delayed > = 1 h/d		1.12 (1.01–1.23)	0.026
Weekend wake time			
Did not deviate		1.00	
Delayed < 1 h/d		0.99 (0.91–1.09)	0.880
Delayed >= 1 h/d		1.11 (1.01–1.22)	0.026
Social jetlag			
< 1 h		1.00	
>= 1 h		1.06 (0.99–1.14)	0.100

Note: adjusted for age, sex, family income, parental education level, parental myopia, academic record, and academic workload.

addition, at the symposium of the European Academy of Optometry and Optics in Dublin in 2022, a poster was defended in which it was concluded that myopic children had more variability in sleep than non-myopic children and that the greater the spherical myopic equivalent, the worse the quality of sleep [34].

The precise mechanisms involved in the association between sleep duration, sleep-wake schedule, and myopia risk in children and adolescents have not been fully understood. An irregular sleep-wake schedule is a manifestation of circadian rhythm disorder [6]. Circadian rhythms exist in axial length, choroidal thickness, and intraocular pressure [35]. Under normal circadian rhythms, the axial length is the longest during the day and the shortest at night, while the choroid is thickest at night and thinnest during the day [36]. When sleep deprivation causes disturbance to the circadian rhythm, the axial daily rhythm and the choroidal daily rhythm undergo phase shifts, leading to myopia [37]. Moreover, neuroendocrine may influence the development of myopia. When light stimulates the retina, photoreceptor signals initiate dopamine release from secretory cells and inhibit hormone synthesis in the pineal gland by integrating inherently light-sensitive retinal ganglion cells sensitive to short wavelengths [38]. Bedtime exposure to high light levels activates the retinal dopaminergic pathway through signaling from retinal ganglion cells, which leads to axial elongation of the ocular axis [29]. Myopia may also be related to increased endogenous melatonin production. According to a recent study, myopes had significantly shorter sleep duration and higher night-type diurnal preference than emmetropes (all *P* < 0.05), and their urinary melatonin levels were significantly lower (29.17 ± 18.67) than emmetropes (42.51 ± 23.97, *P* = 0.04, *d* = 0.63) [39]. In addition, an experimental study revealed that exposure to room light in the late evening suppresses the start of melatonin synthesis and shortens the duration of melatonin production [40]. More research is needed to explore the causes and potential influencing factors of myopia in children and adolescents.

Our findings suggest that investigating and understanding irregular sleep-wake schedules in modern society could be an effective approach to understand and prevent myopia. Prevention and control of myopia in pediatric and adolescent populations should focus not only on ensuring adequate sleep but also on maintaining a regular sleep-wake schedule. The findings in this report are subject to at least three limitations. First, myopia data were obtained by self-reporting and were not confirmed by cycloplegia and computer refraction. Second, this study investigated participants in the Baoan District of Shenzhen City and the data cannot be generalized. Third, the cross-sectional study can only demonstrate a correlation between sleep duration, sleep-wake schedule, and myopia, but cannot determine the causal relationship between them. Longitudinal cohort studies or randomized controlled trials using objective sleep measures of community-based sleep education intervention should be conducted to further clarify the etiology of myopia.

## Data Availability

The data presented in this study is available on request from the corresponding author.
